# Clinical efficacy and safety of sintilimab injection plus chemotherapy in patients with stage IB–IIIB non-small cell lung cancer

**DOI:** 10.12669/pjms.41.3.9830

**Published:** 2025-03

**Authors:** Qiang Guo, Haijun Wang, Yanzhao Xu, Mingbo Wang, Ziqiang Tian

**Affiliations:** 1 Qiang Guo Department of Thoracic Surgery, Affiliated Hospital of Hebei University, Baoding 071000, Hebei, China; 2 Haijun Wang Department of Thoracic Surgery, Xingtai People’s Hospital, Xingtai 054000, Hebei, China; 3 Yanzhao Xu Department of Thoracic Surgery, The Fourth Hospital of Hebei Medical University, Shijingshan 050000, Hebei, China; 4 Mingbo Wang Department of Thoracic Surgery, The Fourth Hospital of Hebei Medical University, Shijingshan 050000, Hebei, China; 5 Ziqiang Tian Department of Thoracic Surgery, The Fourth Hospital of Hebei Medical University, Shijingshan 050000, Hebei, China

**Keywords:** Sintilimab injection, Non-small cell lung cancer, Injectable albumin-Bound Paclitaxel, Clinical Efficacy

## Abstract

**Objective:**

To investigate the clinical efficacy of sintilimab injection and chemotherapy as neoadjuvant therapy for stage IB–IIIB non-small cell lung cancer(NSCLC), and evaluate its clinical safety.

**Methods:**

A retrospective analysis was conducted on the clinical data of patients with stage IB–IIIB NSCLC who underwent neoadjuvant treatment before surgery at Affiliated Hospital of Hebei University, between June 2021 to August 2023. Eighty patients were divided into an observation group(n = 40) and a control group(n = 40) according to their treatment regimens. The control group was administered albumin-bound paclitaxel plus cisplatin, while the observation group received sintilimab injection in addition to the exact treatment provided for the control group. The clinical efficacy, tumor marker levels, immune function, and quality of life were compared between the two groups.

**Results:**

After treatment, the overall response rate in the observation group was 85.00%, significantly higher than 65.00% in the control group(P< 0.05). The overall pathological response rate in the observation group was 77.5%, significantly higher than 42.5% in the control group (P< 0.05).During treatment, adverse reactions were observed in both groups. Yet, there was no statistically significant difference in the incidence of adverse drug reactions between the two groups (P > 0.05).

**Conclusion:**

The combination therapy of sintilimab injection, albumin-bound paclitaxel, and cisplatin for stage IB–IIIB NSCLC can effectively reduce tumor marker levels, improve immune function, demonstrating favorable clinical and pathological efficacy without increasing the incidence of adverse drug reactions.

## INTRODUCTION

In 2020, there were approximately 19.3 million new cancer cases and around 10 million new fatalities globally, with lung cancer having the second highest incidence (11.4%) and ranking the top among fatal diseases, comprising 18% of total cancer-related deaths.^1^ Non-small cell lung cancer(NSCLC) is the most commonly occurring lung cancer, accounting for 80%–85% of diagnoses. In China, statistics show that lung cancer has the highest incidence and mortality rates among malignant tumors.^2^

NSCLC has an insidious onset, with early stages exhibiting few discernible clinical symptoms. Consequently, many patients are diagnosed at advanced stages, where surgical intervention alone yields limited efficacy. Preoperative and postoperative chemotherapy has been proven to shrink lesions and improve prognosis to some extent. Currently, immunotherapy for malignant tumors has emerged to be a research hotspot, with diverse immunomodulators finding widespread clinical application. Evidence has shown that programmed cell death protein 1/death ligand 1(PD-1/PDL-1) signaling pathways play pivotal roles in tumor evasion during NSCLC progression, and when used as adjunctive therapy for NSCLC, PD-1 inhibitors can increase tumor suppression rates.^3^ Sintilimab, a PD-1 monoclonal antibody and novel PD-1 inhibitor, exhibits a high affinity for PD-1, thus achieving high, stable target occupancy. For this reason, sintilimab is frequently used in clinical practice.^4^

Sintilimab injection, a domestically developed immune-targeted drug approved by the National Medical Products Administration (NMPA) of China, shows proven clinical efficacy in numerous clinical trials across various malignant tumors. On this basis, this study investigated the therapeutic effects of sintilimab injection combined with chemotherapy on patients with stage IB–IIIB NSCLC, and evaluated the clinical efficacy and safety of this combination therapy in comparison with albumin-bound paclitaxel plus cisplatin as monotherapy, aiming to provide insights for the clinical management of stage IB–IIIB NSCLC.

## METHODS

This was a retrospective study. A total of 80 patients who were diagnosed with stage IB–IIIB NSCLC and underwent surgery following neoadjuvant therapy at the Department of Thoracic Surgery, Affiliated Hospital of Hebei University, between June 2021 to August 2023 were included in this study. They were divided into an observation group(n = 40) and a control group(n = 40) According to different treatment regimens.

### Ethical Approval:

The study was approved by the Institutional Ethics Committee of Affiliated Hospital of Hebei University (No.: HDFYLL-KY-2023-082; Dated: June 19, 2023), and written informed consent was obtained from all participants.

### Inclusion criteria:

- NSCLC cases meeting the diagnostic criteria outlined in the *Chinese Medical Association Guideline for Clinical Diagnosis and Treatment of Lung Cancer (2023 Edition)* and confirmed by histopathological biopsy.^5^
- Patients between 45 and 75 years of age.
- NSCLC classified as stage IB to IIIB as defined by the tumor, node, metastasis(TNM) staging system.
- Estimated survival ≥ 6 months.
- Fully conscious state.
- Availability of complete clinical data.
- Patients and their families who had good compliance with treatment and were willing and able to cooperate with the completion of this study.

### Exclusion criteria:

- Presence of other malignant tumors.
- Inability to tolerate chemotherapy and/or sintilimab treatment.
- Incomplete clinical data.

### Treatment Regimens:

Patients in the control group received treatment with albumin-bound paclitaxel plus cisplatin as follows: albumin-bound paclitaxel injection was administered intravenously at a dose of 260 mg/m², followed by cisplatin at a dose of 30 mg/m² via the intravenous route on days one to three. Each cycle of treatment lasted 21 days In the observation group, sintilimab injection was administered intravenously at a dose of 200 mg on the first day of chemotherapy, followed by albumin-bound paclitaxel injection on the second day, which was administered in the exact same way as in the control group. Cisplatin was administered intravenously at a dose of 30 mg/m² on days two to four. Each cycle of treatment lasted 21 days. Both groups of patients underwent three cycles of treatment before surgery, followed by a six-month follow-up period postoperative. During the course of chemotherapy, patients received fluid infusion as appropriate and prompt management of adverse reactions. All operations were performed by the same group of doctors.

### Outcome Measures:

### Clinical Efficacy:

Clinical efficacy was evaluated according to the criteria set forth by the Union for International Cancer Control (UICC) and the World Health Organization (WHO):^6^ A complete response (CR) is defined as complete disappearance of all tumor lesions, with sustained absence lasting for at least one month; a partial response (PR) represents a decrease in tumor size by at least 50%, with no disease progression and the condition staying stable for at least one month without the occurrence of new lesions; stable disease (SD) means a decrease in tumor size by less than 50% or a slight increase in tumor size by less than 25%, with the condition lasting for at least one month; progressive disease (PD) indicates an increase in tumor size by over 25% or presence of new lesions. The overall response rate (ORR) was calculated as follows: ORR = (Number of CR cases + Number of PR cases) / Total number of cases × 100%

### Pathological Response:

Surgical specimens were evaluated according to the Expert Consensus on the Pathological Evaluation of Neoadjuvant Therapy Efficacy for Non-small Cell Lung Cancer:^7^ a major pathological response (MPR) is defined as < 10% residual viable tumor cells in the tumor bed after treatment, regardless of the presence of residual viable tumor cells in lymph nodes; a pathological complete response (pCR) means 0% residual viable tumor cells in the tumor bed and lymph nodes after treatment. The overall pathological response rate (OPRR) was calculated as follows: OPRR = (Number of MPR cases + Number of pCR cases) / Total number of cases × 100%.

### Immune Function:

Peripheral blood samples (3 mL) were collected from both groups of patients on an empty stomach before treatment and at six months post-treatment. After centrifugation at 4000 rpm (radius: 5 cm) for 10 minutes, the plasma on top was harvested. Flow cytometry was conducted to determine the levels of CD3+ and CD4+, as well as the CD4+/CD8+ ratio. Quantitative nephelometry was employed to measure the levels of immunoglobulin M (IgM), immunoglobulin G (IgG), and immunoglobulin A (IgA).

### Tumor Markers:

Venous blood samples were collected from both groups of patients on an empty stomach before treatment and at six months post-treatment. Carcinoembryonic antigen (CEA), neuron-specific enolase (NSE), and vascular endothelial growth factor (VEGF) levels were measured using electrochemiluminescence immunoassay (ECLIA).

### Quality of Life and Exercise Tolerance:

The Chinese version of the Functional Assessment of Cancer Treatment-Lung (FACT-L) Scale was utilized to evaluate the quality of life of patients in both groups before and after treatment.^8^ This scale comprises 36 items across two dimensions, with lower scores indicating poorer quality of life. Exercise tolerance was evaluated using the 6-Minute Walk Test.^9^

### Adverse Drug Reactions:

The National Cancer Institute Common Toxicity Criteria version 3.0 (NCICTC 3.0) was adopted to evaluate the adverse reactions caused by the treatment regimens.^10^ Adverse drug reactions included bone marrow depression, gastrointestinal discomfort, fatigue, rash/hair loss, and hepatic/renal function impairment.

### Statistical analysis:

The software SPSS20.0 was used for statistical analysis. The confidence interval was 95%. Enumeration data were presented as “frequency (percentage) [*n*(%)]” and intergroup comparisons were examined by the chi-square (*χ*²) test. Measurement data were expressed as “mean ± standard deviation (*X̅±S*)” and intergroup comparisons were analyzed using independent sample t-tests. *P*< 0.05 was considered statistically significant.

## RESULTS

The observation group consisted of 40 patients, including 24 males and 16 females, with an age range of 45 to 75 years and a mean age of (56.30 ± 7.11) years. The control group comprised 40 cases, including 22 males and 18 females aged between 42 and 76 years, with a mean age of (57.33 ± 7.65) years. There were no statistically significant differences in sex, age, or TNM stage between the two groups (all *P* > 0.05), indicating high comparability (Table-I). After treatment, the observation group exhibited an ORR of 85.00%, significantly higher than 65.00% in the control group (*P* < 0.05). In terms of pathological response, the OPRR in the observation group was 77.50%, significantly higher than 42.50% in the control group (*P* < 0.05) (Table-II).

**Table-I:** Comparison of general patient characteristics between the two groups.

Item	Observation group (n = 40)	Control group (n = 40)	t/χ²	P
Age (years)	56.30±7.11	57.33±7.65	0.620	0.537
Sex (male/female, n)	24/16	22/18	0.205	0.651
***TNM stage***			0.687	0.953
IB	5	4		
IIA	3	4		
IIB	6	6		
IIIA	15	16		
IIIB	11	10		

**Table-II:** Comparison of clinical efficacy and pathological response between the two groups [*n*(%)].

Group	n	CR	PR	SD	PD	MPR	pCR
Observation group	40	4(10.00)	30(75.00)	5(12.50)	1(2.50)	8(20.00)	23(57.50)
Control group	40	2(5.00)	24(60.00)	12(30.00)	2(5.00)	13(32.50)	4(10.00)
*χ^2^*		8.632				4.381	
*P*		0.035				0.036	

Before treatment, the two groups showed no significant differences in CD3+, CD4+, CD4+/CD8+, IgM, IgG, and IgA levels (all *P* > 0.05). After treatment, the levels of CD3+, CD4+, CD4+/CD8+, IgM, IgG, and IgA in both groups showed significant improvement (*P* < 0.05, respectively), and the improvement was significantly more pronounced in the observation group than in the control group (*P* < 0.05, respectively) (Table-III).

**Table-III:** Comparison of pre- and post-treatment immune function between the two groups (*χ̅*±*S*).

Outcome measure	Time point	Observation group	Control group	t	P
CD3^+^ (%)	Before treatment	42.71±6.31	42.88±6.17	0.122	0.903
	After treatment	48.63±6.23	43.33±6.10	3.839	0.000
CD4^+^ (%)	Before treatment	27.32±4.14	27.45±3.82	0.146	0.884
	After treatment*	38.19±4.56	33.99±4.68	4.065	0.000
CD4^+^/CD8^+^ (%)	Before treatment	1.28±0.10	1.27±0.07	0.500	0.619
	After treatment*	1.75±0.18	1.55±0.15	5.567	0.000
IgM (g/L)	Before treatment	1.39±0.65	1.38±0.71	0.013	0.990
	After treatment*	2.77±0.64	2.42±0.52	2.708	0.008
IgG(g/L)	Before treatment	1.36±0.27	1.33±0.31	0.416	0.679
	After treatment*	2.86±0.76	2.32±0.48	3.778	0.000
IgA (g/L)	Before treatment	1.35±0.27	1.32±0.31	0.444	0.658
	After treatment*	2.84±0.75	2.24±0.42	4.392	0.000

Before treatment, there were no statistically significant differences in CEA, NSE, and VEGF levels between the two groups (all *P* > 0.05). After treatment, the levels of CEA, NSE, and VEGF in both groups decreased significantly (*P* < 0.05, respectively). Moreover, the reduction in tumor marker levels was significantly more noticeable in the observation group compared to the control group (*P* < 0.05, respectively) (Table-IV).

**Table-IV:** Comparison of tumor marker levels between the two groups (*χ̅*±*S*).

Tumor marker		Observation group	Control group	t	P
CEA (ng/ml)	Before treatment	14.11±3.29	14.28±3.47	0.218	0.828
	After treatment	5.88±1.03	7.09±1.24	4.741	0.000
NSE (ng/ml)	Before treatment	46.08±7.21	46.28±6.99	0.128	0.899
	After treatment	21.37±5.29	27.72±5.70	5.164	0.000
VEGF (ng/ml)	Before treatment	224.53±22.58	225.77±23.76	0.238	0.813
	After treatment	133.80±4.48	146.55±5.07	11.914	0.000

Before treatment, the two groups did not differ greatly in the FACT-L score and 6-minute walk distance (6MWD) (all *P* > 0.05). After treatment, both the FACT-L score and 6MWD in both groups showed significant improvement (*P* < 0.05, respectively). Notably, the improvement in the observation group was significantly more pronounced compared to the control group (*P* < 0.05, respectively) (Table-V). During treatment, patients in both groups experienced adverse reactions. However, the two groups showed no significant differences in the incidence rates of bone marrow depression, gastrointestinal discomfort, fatigue, hepatic/renal function impairment, and rash/hair loss (all *P* > 0.05) (Table-VI).

**Table-V:** Comparison of quality of life and exercise tolerance between the two groups (*χ̅*±*S*).

Group	FACT-L score		6MWD (m)	

	Before treatment	After treatment	Before treatment	After treatment
Observation group	76.95±6.92	90.23±5.57	383.20±8.67	346.58±6.79
Control group	77.30±6.92	82.45±6.88	382.48±8.60	355.63±7.28
*t*	0.226	5.556	0.376	5.751
P	0.822	0.000	0.708	0.000

**Table-VI:** Comparison of adverse reactions between the two groups [*n*(%)].

Group	Bone marrow depression	Gastrointestinal discomfort	Fatigue	Hepatic/Renal function impairment	Rash/Hair loss
Observation group	14(35.00)	17(42.50)	8(20.00)	16(40.00)	9(22.50)
Control group	15(37.50)	14(35.00)	6(15.00)	13(32.50)	6(15.00)
*c* ^2^	0.054	0.474	0.346	0.487	0.738
*P*	0.816	0.491	0.556	0.485	0.390

## DISCUSSION

The results of this study demonstrate that clinically, the ORR and OPRR in the observation group treated with sintilimab in combination with albumin-bound paclitaxel plus cisplatin were significantly higher than those in the control group using albumin-bound paclitaxel plus cisplatin alone (*P*< 0.05), aligning with the findings of previous studies. Sintilimab has unique binding epitopes on PD-1, and the PD-1/PD-L1 axis has recently emerged as a novel target for immunotherapy in cancer treatment.^11^ By binding to PD-1, sintilimab blocks the interaction between PD-1 and its ligands PD-L1 and PD-L2, resolves immune suppression, enhances T cell-mediated immune surveillance, and inhibits tumor cell proliferation, thus achieving effective disease control.^12^ T cell subsets play an essential role in cellular immune response.

The levels of T cell differentiation subsets in patients with malignant tumors are lower than in healthy individuals.^13^ Immunoglobulins are the primary executors of humoral immune activities, and the levels of IgG, IgA, and IgM are closely associated with humoral immune response.^14^ In summary, both the cellular and humoral immune systems are affected when malignant tumors occur. The results of this study indicate that the improvement in T cell subsets and immunoglobulin levels in the observation group after treatment was significantly more pronounced compared to the control group (*P*<0.05, respectively). This suggests that the combination of sintilimab with albumin-bound paclitaxel plus cisplatin can significantly improve the immune function of patients with stage IB–IIIB NSCLC, consistent with existing evidence.

Detecting tumor marker levels is an important means of diagnosing and evaluating the treatment effectiveness of NSCLC, and it has high patient acceptance and is easy to monitor dynamically.^15^ There are many types of serum tumor markers in NSCLC, among which NSE and CEA are commonly used indicators for NSCLC diagnosis, clinical efficacy, and prognosis evaluation. Research has shown that changes in NSE and CEA levels are related to the pathological stage and malignancy of patients.^16^ Detecting NSE and CEA levels can guide clinical treatment and evaluate treatment efficacy, reflecting changes in the patient’s condition.^17^ The results of this study showed that the levels of NSE and CEA in the observation group patients were significantly reduced after treatment compared to the control group (P<0.05). The combination therapy of Xindilimumab, albumin bound paclitaxel, and cisplatin can reduce the levels of NSE and CEA in stage IB-IIIB NSCLC, and the clinical effect is good. This is consistent with multiple research findings.^12^,^18^ Angiogenesis runs through the entire process of NSCLC occurrence and development. VEGF, as an important promoting factor in angiogenesis, plays a crucial role in promoting tumor tissue angiogenesis and dilation, and plays an important role in the occurrence and development of NSCLC patients. The observation group in this study showed a significant decrease in VEGF levels after treatment compared to the control group (P<0.05), indicating that the combination of Xindilimumab, albumin bound paclitaxel, and cisplatin in the treatment of stage IB - III B NSCLC can reduce VEGF levels and inhibit tumor cell proliferation.

The improvement of quality of life for patients with malignant tumors and the adverse reactions to treatment are increasingly valued. Good treatment outcomes, lower incidence of adverse reactions, and better quality of life for patients are the main aspects of evaluating treatment. Multiple studies have shown that xindilimab has ideal control effects on advanced NSCLC without increasing adverse reactions.^19^,^20^ The results of this study showed that the quality of life score and exercise endurance test score of the observation group were higher than those of the control group after treatment (P<0.05), and the incidence of adverse reactions was comparable to that of the control group (P>0.05). The combination of Xindilimumab and albumin bound paclitaxel in the treatment of stage IB-IIIB NSCLC can improve the patient’s overall health status, enhance their quality of life, and not increase adverse reactions. This is consistent with previous research findings.

### Limitations:

It includes a small sample size and a short follow-up period. In the future, studies with a larger sample size and an extended follow-up period are warranted to further evaluate the clinical efficacy of sintilimab combined with albumin-bound paclitaxel plus cisplatin in treating stage IB–IIIB NSCLC.

## CONCLUSIONS

The combination of sintilimab with albumin-bound paclitaxel plus cisplatin for stage IB–IIIB NSCLC treatment demonstrates favorable clinical efficacy and safety and achieves significant improvement in quality of life and exercise tolerance, thus worthy of clinical promotion and application.

### Authors’ Contributions:

**QG** and **ZT:** Carried out the studies, participated in collecting data, and drafted the manuscript, and are responsible and accountable for the accuracy or integrity of the work.

**HW:** Literature search**,** Performed the statistical analysis and participated in its design.

**YX** and **MW:** Participated in acquisition, analysis, or interpretation of data and draft the manuscript.

All authors read and approved the final manuscript.
